# The olfactory bulb is a source of high-frequency oscillations (130–180 Hz) associated with a subanesthetic dose of ketamine in rodents

**DOI:** 10.1038/s41386-018-0173-y

**Published:** 2018-08-08

**Authors:** Mark Jeremy Hunt, Natalie E Adams, Władysław Średniawa, Daniel K Wójcik, Anna Simon, Stefan Kasicki, Miles Adrian Whittington

**Affiliations:** 1 University of York, Heslington, York, YO10 5DD UK; 2 Nencki Institute of Experimental Biology, 3 Pasteur Street, Warsaw, 02-093 Poland; 3 Faculty of Biology, University of Warsaw, Miecznikowa 1, Warsaw, 02-096 Poland

**Keywords:** Schizophrenia, Neurophysiology

## Abstract

High-frequency neuronal population oscillations (HFO, 130–180 Hz) are robustly potentiated by subanesthetic doses of ketamine. This frequency band has been recorded in functionally and neuroanatomically diverse cortical and subcortical regions, notably ventral striatal areas. However, the locus of generation remains largely unknown. There is compelling evidence that olfactory regions can drive oscillations in distant areas. Here we tested the hypothesis that the olfactory bulb (OB) is a locus for the generation of HFO following a subanesthetic dose of ketamine. The effect of ketamine on the electrophysiological activity of the OB and ventral striatum of male Wistar rats was examined using field potential and unit recordings, local inhibition, naris blockade, current source density and causality estimates. Ketamine-HFO was of larger magnitude and was phase-advanced in the OB relative to ventral striatum. Granger causality analysis was consistent with the OB as the source of HFO. Unilateral local inhibition of the OB and naris blockade both attenuated HFO recorded locally and in the ventral striatum. Within the OB, current source density analysis revealed HFO current dipoles close to the mitral layer and unit firing of mitral/tufted cells was phase locked to HFO. Our results reveal the OB as a source of ketamine-HFO which can contribute to HFO in the ventral striatum, known to project diffusely to many other brain regions. These findings provide a new conceptual understanding on how changes in olfactory system function may have implications for neurological disorders involving NMDA receptor dysfunction such as schizophrenia and depression.

## INTRODUCTION

Ketamine is a non-competitive NMDA antagonist that is used widely to model acute psychoses in humans and experimental animals [1] and more recently to treat depression [2]. NMDAR antagonists can affect neuronal oscillations, including gamma and delta rhythms, both associated with schizophrenia and depression [3–5]. In rodents, a prominent feature observed after subanesthetic injection of ketamine, and related NMDAR antagonists, are high-frequency oscillations (HFO, 130–180 Hz) in field potential recordings from the ventral striatum (VS), caudate, hippocampus, diverse cortical and brainstem regions [6–10]. There is evidence that VS subregions are involved, at least in part, in ketamine-HFO generation [11], however, to date, the origin of this widespread oscillation remains unclear.

Elevated HFO has also been reported in serotonergic [12] and neurodevelopmental rodent models of schizophrenia [10, 13] but it is unclear if this rhythm is altered in patients. Certainly, the HFO-frequency range overlaps with what some researchers call ‘high-gamma’ rhythms which are implicated widely in sensory processing in humans [14]. In rodents, HFO as part of physiological hippocampal sharp waves, are well known to mediate memory consolidation [15], indicating abnormal HFO rhythms may influence fundamental cognitive processes.

Oscillatory activity in olfactory systems, in particular the olfactory bulb (OB), can potently drive coherent rhythmic activity in anatomically distant brain areas [16, 17]. One of these putative target areas is the VS, which, in turn, is reciprocally connected to many brain regions involved in emotion and motivation [18]. Although not widely associated with psychiatric disease, disturbances in olfactory function can be the earliest symptoms manifest in patients with schizophrenia, often occurring during the preceding at-risk phase [19]. Olfactory dysfunction is also becoming increasingly recognised as a prodromal marker for neurodegenerative disorders [20]. Additionally, in rodents, primary disruption of olfactory processing leads to behaviours that are unrelated to olfactory deficits involving cortico-limbic networks [21]. A high-frequency rhythm (100–130 Hz) in the rodent OB has been reported under ketamine–xylazine anaesthesia indicating OB circuitry can generate fast rhythms [22]. The need to clearly identify unambiguous sources of ketamine-HFO, and the limited number of studies investigating how ketamine influences olfactory networks, prompted us to investigate the effect of ketamine on LFP oscillations in the OB and their possible impact on the nucleus accumbens (NAc) subregion of the VS.

## MATERIALS AND METHODS

### Surgery and recording

Twenty male Wistar rats (250–350 g) were used in this study. In nine rats, tungsten electrodes (125 µm, Science Products, Germany) were implanted bilaterally in OB (AP + 7.5, ML = ±0.5, DV = 3–3.5 mm) and in the right VS (AP = +1.6, ML = +0.8, DV = 7 mm) [23]. In seven rats, 22 gauge stainless steel guides (Bilaney, Germany) and electrodes were implanted bilaterally in the OB and electrodes bilaterally in the VS. In four rats a microdrive-mounted 32-channel silicon probe (Edge-10 mm-20-177-H32_21 mm, Neuronexus, USA), interelectrode distance 20 µm, was implanted in the right OB. A screw posterior to the bregma was used as a reference/ground.

One week after surgery, rats were placed in an arena (50×50×50 cm) and local field potentials (LFPs) recorded before and after 25 mg/kg ketamine injection (i.p). LFP’s were amplified ×1000, filtered 0.01–1000 Hz and digitised at 1.6 kHz (AlphaLab, AlphaOmega, Israel). A number of other studies have examined the effect of other doses of ketamine (e.g. 10–60 mg/kg, [24]) on LFP oscillations in other frequency bands in rodents (<90 Hz), however we, and others have found that 25 mg/kg produces the most robust increase in HFO power [8]. Intraperitoneal saline injection was used as a control for systemic ketamine injection in 6 rats. Infusions: 0.5 µg muscimol (0.5 µl/1-min) was infused (28 gauge needle, Bilaney, Germany) to one OB and saline to the other side. Rats were reconnected and 25 mg/kg ketamine injected (i.p) immediately. At least 72 h later the experiments were repeated with muscimol/saline-infused sides reversed. Naris blockade: These experiments were carried out during the recovery phase of ketamine anaesthesia 200 mg/kg. During recovery, rats are manageable, and naris blockade can be achieved in a straightforward manner. Importantly, during recovery HFO is visible in LFP spectra from VS [8] and OB (Supplementary Figure 2 and Fig. 3). A cotton bud was used to press a soft rubber base to close one naris (at least 60 s). This procedure was repeated for the opposite naris. Rats for this study were also used in the systemic ketamine study. Multi-unit activity (MUA): Rats chronically implanted with silicon 32-channel probes which were gradually advanced over several recording days until they reached a depth of around 3–4 mm or phase reversal was visible. Wideband signals were recorded from freely moving rats (DC-11 kHz, sampled at 44 kHz). Recordings were made before and after injection of ketamine 25 mg/kg at different depths with 3–4 days separating each recording session. Electrode locations were determined on 40 µm Cresyl violet (Sigma, UK) or Hoechst (Sigma, UK) stained sections. All experiments were conducted in accordance with the European community guidelines on the Care and Use of Laboratory Animals (86/609/EEC) and approved by a local ethics committee.

### Analysis

Mean power spectra of the LFP were carried out on successive 60-s data blocks using a fast Fourier transform (4096 points) and the dominant power of HFO (130–180 Hz) calculated (Spike 2, CED, UK). Granger causality was computed over a series of orders (1–10) for 1–350 Hz. LFPs were split into 500 ms sections overlapped by 10 ms to create trials over which results were averaged, using a 50 ms window with one_bi_ga.m from the BSMART toolbox (SHIS UT-Houston, USA), MATLAB. Coherence spectra (2048 points) were computed for OB and VS, using the raw LFP, for 60 s before and after ketamine injection. Waveform correlation between the signals was computed using the 130–180 Hz band-pass filtered signal over 60 s. Current source density (CSD) analysis of the 130–180 Hz filtered signal was performed with the kernel CSD method [25]. Average CSD’s for each rat were triggered on troughs of HFO in the most dorsal electrode. Phase differences were calculated for the 130–180 Hz filtered signal with respect to the most dorsal contact (where HFO has large amplitude). MUA was extracted using a threshold of 1.5 or 3 SD of the 500 Hz high-pass Butterworth third order filtered signal. Negative peaks of spikes were marked as events. HFO troughs of the most dorsal contact were used to trigger MUA (non-overlapping 50 ms sweeps).

### Statistical analysis

Unless stated otherwise, data were analysed using repeated-measures ANOVA followed by the Bonferroni post hoc test. *p* < 0.05 were considered statistically significant.

## RESULTS

### The OB leads ketamine-HFO in ventral striatal areas

**Fig. 1:**
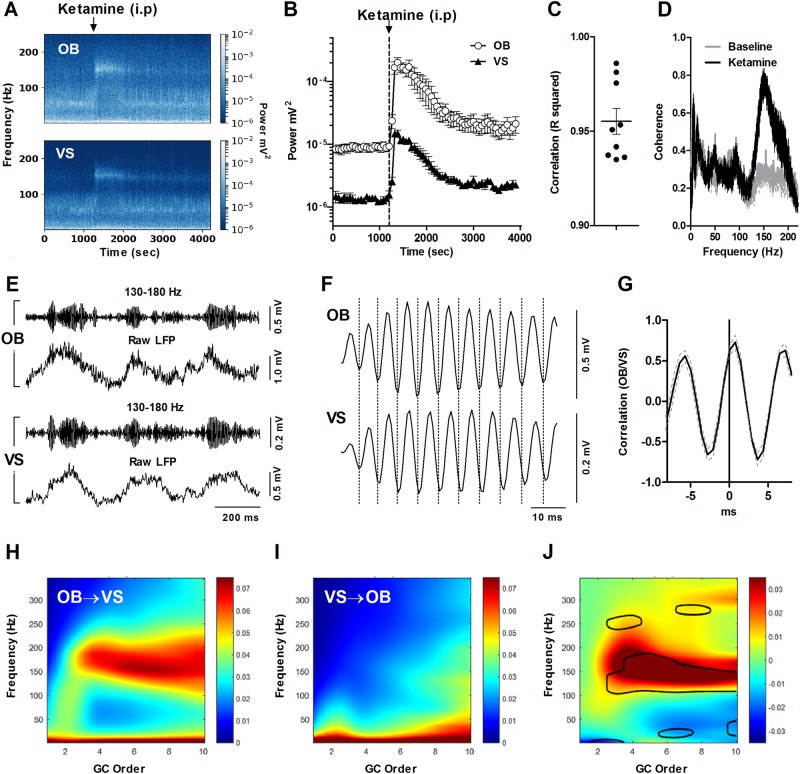
Ketamine-enhanced HFO in the OB precedes the VS. **a** LFP spectrograms of simultaneous recordings in the OB and VS showing the effect of intraperitoneal injection of 25 mg/kg ketamine. Note the strong power of HFO (close to 150 Hz) immediately after injection of ketamine. The same scale is used for both plots. **b** Time-course showing mean ± SEM HFO power in the OB (white circles) and VS (black triangles) before and after injection of ketamine (*n* = 9 rats). The power of HFO was significantly larger in the OB. **c** Correlation of HFO power in the OB and VS was highly significant for all rats (each data point represents an individual rat). The mean ± SEM is also shown. **d** Ketamine injection was associated with an increase in the mean coherence between the OB and VS, which was prominent at the HFO band. **e** 130–180 Hz band-pass filtered signal (top pair) and the raw LFP (bottom pair) recorded simultaneously from the OB and VS shortly after injection of ketamine. Bursts of HFO are clearly visible in recordings from both sites. **f** Expansion of a single HFO burst showing that HFO recorded in the OB precedes the VS. Dotted lines indicate the troughs of HFO in the OB. **g** Mean waveform correlation for the 130–180 Hz band-pass filtered signal in the VS relative to the OB. Zero is the peak of the OB HFO waveform showing HFO occurs relatively later in the VS. **h**, **i** Mean Granger causality relationship for the OB → VS and VS → OB direction, respectively. **j** Mean difference between the Granger analyses the contoured areas indicate significant differences

Intraperitoneal injection of 25 mg/kg ketamine produced almost immediate overt behavioural activation (characterised by hyperlocomotion, ‘circling’ and rearing) of 10–15 min duration which correlates with increases in the power of HFO [8]. Figure 1 shows the effect of intraperitoneal 25 mg/kg ketamine administration on the power of HFO recorded in the OB and VS. Increased HFO power lasted around 15 min after which time the power gradually returned to baseline values. As can be seen in the representative spectrogram, shown in Fig. 1a, ketamine mainly increased power in the HFO band, though changes in other frequency bands were evident (see below). Quantitative comparison of the group means revealed that HFO power was significantly larger in the OB compared to the VS, both at baseline and after injection of ketamine (brain region × time F64,1024 = 9.82, *p* < 0.0001) (Fig. 1b). This finding was particularly striking, since previous reports have shown the power of HFO in the VS to be larger than in other areas (e.g. dorsal striatum, hippocampus, visual and cortical areas) [6, 11, 26]. Minute-by-minute analysis post ketamine injection revealed a positive correlation (*p* < 0.0001, *n* = 9) between the power of HFO in the OB and VS (*R*^2^ range 0.93–0.98, Fig. 1c). Systemic injection of saline did not influence the power of HFO recorded in the OB (F39,234 = 0.032, *p* = 1.0, one-way ANOVA, see Supplementary 1, *n* = 6 rats).

Coherence, an index of frequency coupling between simultaneously recorded LFP’s, showed a clear increase at the HFO band and was significantly different between the ketamine and baseline conditions in the 141–163 Hz range (*p* < 0.001, Bonferroni test) (Fig. 1d). Generally, coherence at other frequencies was not different after ketamine injection although a significant reduction in coherence was observed around 2 Hz, delta frequency (*p* < 0.001).

To more precisely examine any relationship of HFO events recorded in the OB and VS, the LFP signal was band-pass filtered at 130–180 Hz. At the macroscopic level bursts of HFO in the OB and VS shared almost identical temporal dynamics (Fig. 1e). However, closer inspection of the filtered signal revealed that HFO recorded in the OB preceded the activity recorded in the VS for multiple cycles (Fig. 1f). Global waveform correlation between the OB and VS revealed a strong correlation with a delay of around 0.45 ± 0.16 ms in the VS (Fig. 1g). This apparent unidirectional relationship between HFO in OB and VS was analysed further: Granger causality estimates can be used to determine the magnitude and direction of the temporal relationship between simultaneously recorded LFPs. The unfiltered LFP Granger causality spectra revealed a strong causality score for the HFO band in the OB → VS direction. In contrast, there was no causality score, above baseline for the HFO band in the VS → OB direction (Fig. 1h, i). Mean difference plots highlighted this HFO-specific, unidirectional pattern of causality in a significant manner (Fig. 1j).

### Normal OB function is required for ketamine-induced HFO in the ventral striatum

**Fig. 2:**
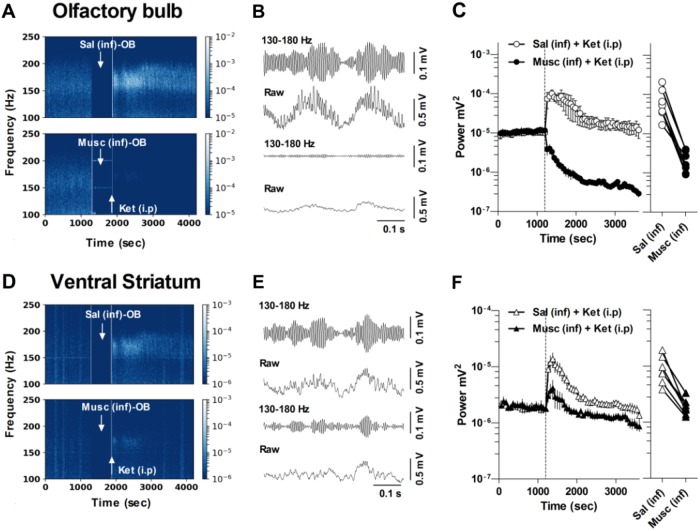
Unilateral muscimol inactivation of the OB attenuates HFO power locally and in the VS on the ipsilateral side. **a**, **d** LFP spectrograms showing the effect of pre-infusion of saline (right OB) or muscimol (0.5 µg/0.5 µl) (left OB) on the power of 25 mg/kg ketamine-enhanced HFO recorded locally in the OB and in the VS. Rats were disconnected for infusion and injection, indicated by the blank spectral spaces in the spectrograms. **b**, **e** Examples of 130–180 Hz band-pass filtered signals and corresponding raw LFP after systemic injection of ketamine for saline (top pair) and muscimol-infused sides (bottom pair). **c**, **f** Mean power of HFO recorded in the OB and VS for muscimol (filled) and saline-infused sides (open), before and after 25 mg/kg ketamine (*n* = 6 rats, two experiments per rat). Ketamine injection is indicated by the dotted line. Adjacent plots show the values for individual rats recorded in the OB and VS after muscimol infusion and ketamine (i.p). Note, unilateral OB-muscimol infusion almost completely attenuated HFO power in the OB, but only partial reduction in power in the VS

To investigate whether OB activity influences HFO recorded in the VS, we implanted rats (*n* = 7) bilaterally with a guide/electrode complex in the OB and recording electrodes in the VS. Within animal design experiments were carried out for each rat, so that muscimol was infused to the left or right OB on different experimental days. Saline was infused to the contralateral OB as a control and the mean of both experiments/rat were calculated. Representative spectrograms of the time-course of ketamine-HFO in the OB and VS are shown in Fig. 2a, d, respectively. The 130–180 Hz band-pass filtered and raw LFP waveforms post ketamine for the OB and VS are shown in Fig. 2b, e, respectively. Large-amplitude HFO was visible in the saline-infused ‘control’ side, whereas muscimol infusion almost completely attenuated HFO. Group analyses revealed that unilateral infusion of 0.5 µg muscimol to the OB led to a dramatic and almost immediate reduction of ketamine-HFO power in both the OB and VS on the ipsilateral side (muscimol × time F56,672 = 5.24, *p* < 0.0001) (Fig. 2c, f). In contrast, increases in HFO power were observed on the saline-infused side. This effect was robust and consistent across all rats (see Fig. 2c, f which show values for individual rats). Of note, unilateral infusion of muscimol to the OB reduced, rather than completely attenuated, the power of ketamine-enhanced HFO in the VS ipsilateral to the infusion (muscimol × time F56,672 = 5.24, *p* < 0.0001).

### Ketamine-HFO are dependent on naris respiratory input

**Fig. 3:**
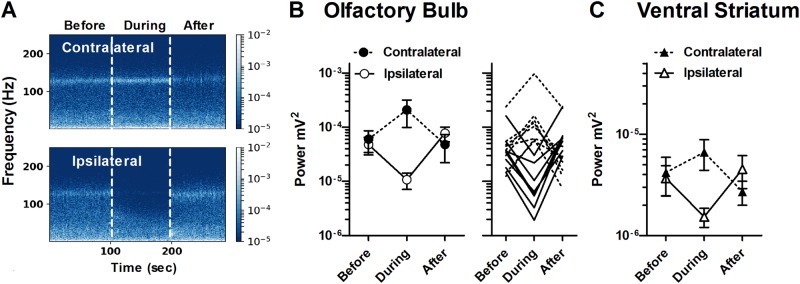
Unilateral naris occlusion reduces HFO power on the ipsilateral side. **a** LFP spectrograms from the OB recorded simultaneously from the side ipsilateral (bottom) and contralateral (top) to naris occlusion. **b** Mean ± SEM power of HFO demonstrating unilateral naris blockade produces an ipsilateral reduction in HFO power and corresponding increase in HFO power on the contralateral side. The adjacent plot shows the power of HFO for individual rats recorded from the contralateral (broken lines) and ipsilateral (continuous lines) sides. **c** Naris occlusion also reduced the power of HFO in the VS

Given the prominent role that the OB appears to have in the generation of HFO we next investigated the effect of naris blockade. These experiments were carried out during recovery from ketamine anaesthesia where HFO begins to emerge (Supplementary Figure 2), rather than in anaesthesia where HFO is absent [8]. At this stage, rats are manageable and short-lasting naris blockade can be carried out. During unilateral naris blockade we observed an almost immediate reduction in the HFO power (repeated-measures one-way ANOVA *F* = 9.6, *p* < 0.0024). Reduction of HFO power in the OB occurred exclusively on the ipsilateral side and quickly recovered when blockade was removed (see example trace in Fig. 3a). In contrast, a tendency for an increase in HFO power was observed on the contralateral side (repeated-measures one-way ANOVA *F* = 3.6, *p* = 0.064). Means of HFO power before, during and after naris blockade are shown in Fig. 3b and individual traces showing the power of HFO from individual rats are also shown. We also analysed the power of HFO in the VS and found parallel changes (Fig. 3c).

### A source of ketamine-HFO lies within the OB

**Fig. 4:**
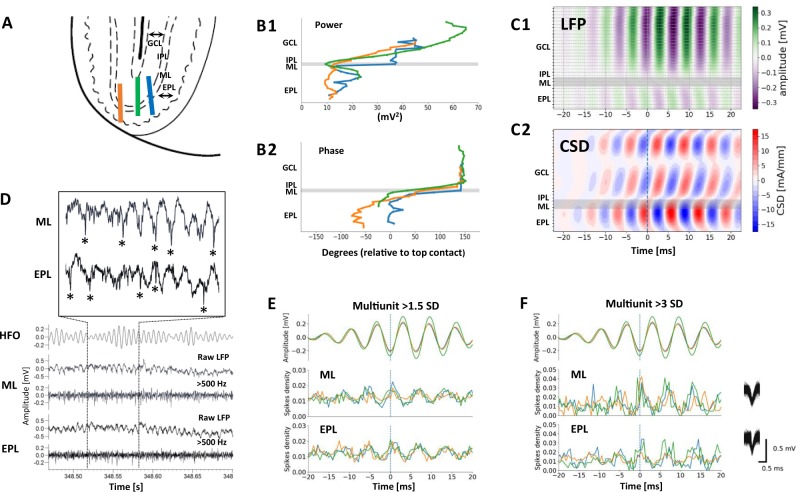
Ketamine-HFO reversal of phase and unit firing occurs close to the ML in the OB. **a** Electrode placements showing the final position of the 32 channels probes implanted in the OB. Each line represents a different rat (*n* = 3 rats); GCL-granule cell layer, IPL-intraplexiform layer, ML-mitral layer, EPL-extraplexiform layer. **b1** HFO power plots showing the power of HFO across the different electrode contacts. **b2** Phase plots (with reference to the most dorsal electrode) showing HFO phase reversal close to the ML. Each colour-coded line corresponds to the electrode placements shown in (**a**). **c1** Example average amplitude plot, for a single rat, triggered on HFO (periods > 3 SD of HFO from the most dorsal contact were used) **c2** CSD analysis of the same data showing HFO dipoles occur close to the ML. **d** Raw, 130–180 Hz band-pass filtered, and multi-unit (>500 Hz) recordings from the ML and EPL. Insert, asterisks indicate the spike-associated HFO in raw LFP’s from the ML and EPL. **e**, **f** Mean discharge probability of multi-unit activity from ML and EPL layers at (global firing was taken at a threshold of >1.5 SD in (**e**) and larger spikes were extracted at a threshold of >3 SD in (**f**). Spike discharge was triggered on the trough of HFO from the most dorsal electrode contact (>3 SD). Multi-unit firing tends to occur close to the negative peak of HFO and on the falling phase of the oscillation on a cycle-by-cycle basis. Values are shown for individual rats (*n* = 3, colour corresponds to implantations shown in **a**)

The laminar distribution of HFO in the OB was investigated in freely moving rats (*n* = 4, one rat excluded due to electrode location) chronically implanted with 32-channel linear silicon probes. Histological examination revealed that the final localisation of the electrodes in three rats spanned the granule cell layer (GCL), intraplexiform (IPL), mitral (ML) and extraplexiform layer (EPL) (Fig. 4a). In one rat histology revealed that the final localisation of the probe was the glomerular and olfactory nerve layer and probe alignment indicated that it did not transect the ML during descent. Consistent with our previous data, marked increases in HFO power were observed after injection of ketamine 25 mg/kg (Supplementary Figure 3).

Analysis of raw LFP’s revealed HFO phase reversal in the three rats where the track transected the ML. We observed a gradual reduction in HFO power moving across the ML (Fig. 4b1). Phase analysis, using the shallowest electrode as a reference, revealed HFO sharply shifted in phase at the level of the ML/IPL (Fig. 4b2). HFO, and the raw LFP remained broadly in phase across all contacts in the implantation that did not transect the ML (excluded rat). CSD analysis revealed clear source/sink pairs close to or at the level of the ML/IPL in the three rats, an example is shown in Fig. 4c along with the corresponding HFO amplitude. No HFO phase reversal was visible when all contacts were above the ML, or in the implantation which did not cross the ML (data not shown).

Spiking was observed in the raw recorded potential (insert in Fig. 4d) which was more pronounced in the IPL/ML and EPL. Global multi-unit activity (MUA), units > 1.5 SD above the mean, was calculated for each rat, which marginally correlated with the negative peak of the field ketamine-HFO recorded at the most distal electrode (Fig. 4e). Isolation of the largest spikes within the MUA (>3 SD above the mean) revealed a strong correlation with the negative peak of the field HFO recorded at the most distal electrode (Fig. 4f). Mitral cells in the ML and tufted cells in the EPL are the principle projection neurons of the OB and thresholded MUA from these two areas were comparably phase-locked to the HFO field for multiple cycles.

## DISCUSSION

The present results reveal a source for ketamine-enhanced HFO in the OB. This HFO effectively projected to VS and was dependent on both neuronal activity within OB and normal naris function. While the precise cellular and network origin of this HFO remains unresolved, large-amplitude spiking in the ML and EPL—containing the main projection neurons in OB—was phase locked to the HFO field.

Axons of OB projection neurons (mitral and tufted cells) are fasciculated and send direct projections to multiple olfactory and non-olfactory regions [27]. This extensive connectivity, which also includes the hippocampus via the entorhinal cortex, may underlie the broad neuroanatomical nature of ketamine-HFO observed in the rat brain. It has long been established that normal oscillatory activity in the rodent OB can be entrained by nasal respiration. For example, air forced in the nostrils of anesthetised rats can produce membrane fluctuations in the OB [28] and air-puffs can reinstate slow OB oscillations in tracheostomised rats [29]. Although we did not measure nasal air flow, ketamine-induced HFO seem to share similarities. They were powerfully attenuated by ipsilateral, but not contralateral, naris blockade. Additionally, HFO recorded after administration of NMDA receptor antagonists are phase amplitude-coupled to slower frequencies, typically delta and theta [7, 30] which tightly align to the so-called 'respiratory rhythm' known to modulate the amplitude of higher frequency rhythms in the other brain areas [31]. It is plausible that increased HFO power in the OB contralateral to naris blockade may be related to compensatory increased respiratory strength, indicating ketamine-HFO may be related to air-flow.

Despite strong OB → VS causality scores, the OB is unlikely to represent the sole source of HFO along this axis. Certainly, the short phase offset (around 0.5 ms) between the OB and VS would rule out volume conduction from OB and render polysynaptic pathways unlikely. Previously, we have observed a similar delay for frontal versus NAc recordings [11]. The physiological explanation for such a short delay (synaptic is typically at least 1 ms) is unclear, however, 0.5 ms is likely to be a conservative estimate since OB recordings were obtained from the GCL, where the power of HFO was larger, rather than the mitral/EPL—the source of the major outputs. Local circuits in many structures have been shown to generate HFO [32, 33]. Indeed, reversible inactivation of the NAc (a subregion of VS) [11] can reduce HFO power locally, and local NMDAR blockade potentiates HFO power [6, 34]. In this study, unilateral reversible inhibition of the OB almost completely attenuated HFO locally, but only reduced HFO power in the VS on the ipsilateral side and with differing temporal dynamics. Together, these findings suggest HFO generators also reside within, or close to, ventral striatal tissue under the influence of the OB generator.

Caution is needed in assigning anatomical sources to LFP oscillations in small structures in vivo. van der Meer and colleagues have shown that gamma oscillations recorded in the VS may arise from volume-conducted signals originating in the piriform cortex [35]. Further, HFO and gamma do not appear to have local source-sink pairs in the VS [26, 35], in line with a more distant source. This lack of definitive localisation of HFO within VS may not have overt interpretational consequences in terms of function though. Although the olfactory tubercle, rather than the NAc receives OB input [36] both ventral striatal areas have been shown to work as a single complex in modulating certain motivational behaviours [37]. In contrast, clear sources and sinks have been observed for HFO (this study) and gamma frequencies in OB [38]. Although microinjection of drugs such as muscimol, used here, may also spread to adjacent structures, the OB is sufficiently anatomically isolated to allow confidence in local action of perfused drugs, especially given the relatively small volume of drug infused. However, we cannot exclude the possibility that large volumes infused to the NAc (see previous paragraph) may also spread to adjacent areas such as the piriform cortex and olfactory tubercle. The role these olfactory regions may play in HFO generation remains to be explored. Further complexity arises when it is considered that local NMDAR blockade in the hippocampus, NAc and prefrontal cortex can also increase the power of HFO locally and in distant areas, indicating possible multiple interconnecting networks may also mediate emergence of this oscillation [6], but again these studies may be confounded by the large volumes and high concentrations of drug used.

Within OB the origin of the HFO remains to be fully resolved. A number of cell subtypes have been shown to generate outputs in this frequency range: Periglomerular interneurons [39], Tufted cells in the EPL [40] and Mitral cells under strong olfactory drive [41]. The dependence on olfactory input, shown by naris occlusion here, has previously been shown to reduce these HFO-frequency neuronal outputs [42]. The network connectivity needed to locally synchronise such neuronal outputs to generate the HFO field are much less clear. Mitral cell-mitral cell excitatory and gap-junctional connectivity is strong but the kinetics of the coupling signals are too slow to support HFO [43, 44]. Shared inhibition onto mitral cells from granule and periglomerular cells also appears far too slow to support HFO, though they can synchronise this cell subtype [45, 46]. However, the rapid bursts of spikes generated by tufted cells have been shown to provide very fast excitatory postsynaptic potentials onto mitral cells [47]. From this we suggest that interaction between an intrinsic mitral cell time-constant and a matching input (perhaps from shared tufted cells)—both compatible with the observed population HFO frequency and both having axons that anatomically converge towards the location of highest HFO field power recorded here (Fig. 4b)—may provide the most parsimonious putative mechanisms for the phenomenon observed.

Granule cells, because of their parallel geometry, are likely to make a major contribution to the LFP signal in the OB [48]. Dendro-dendritic inhibition from granule cells onto mitral cells is important for the synchronisation of mitral cell firing, observed here. In turn, granule cells can be driven by excitatory input from mitral cells and also from the piriform cortex [49]. Although we did not record any obvious spiking in GCL, fast oscillations in the OB can be generated through subthreshold activity of granule cells. Indeed, there is evidence that fast oscillations in the OB, at least for gamma, do not require somatic firing of granule cells [50]. Further studies, possibly using reversible inhibition and in vivo intracellular recordings, are warranted to determine the extent of the neural network underlying HFO after ketamine and the cellular substrate of this oscillation.

The potentiation of HFO via ketamine may be easier to assign a mechanism to. Ketamine selectively targets NMDA receptors on interneurons (NR2A) [51] and thus generates a transient period of disinhibition. Enhanced excitation in the OB shifts mitral cell outputs into the HFO range [41]. Evidence for such an enhanced excitatory drive, via disinhibition, comes from the apparent reciprocal effects of ketamine of HFO and gamma oscillation power (e.g. Fig. 1a). The gamma rhythm is dependent on fast phasic synaptic inhibition [52] and NMDA receptor antagonists have previously been shown to attenuate this frequency band in OB [53] and elsewhere [54].

There is growing evidence for a role for olfaction in psychiatric illness. Neuronal oscillations in olfactory systems are phylogenetically conserved across many species [55] and equivalent circuits are found in humans, sometimes with substantial cross-talk between traditional olfactory areas and those responsible for affective processing. In addition to the evidence cited above it is worth noting that reduced OB volume as well as a shallower olfactory sulcus [19] has been reported in schizophrenia (and in patients at risk). Also, olfactory bulbectomy is one of the most widely employed rodent models of depression [21] and there is growing interest in intranasal delivery of ketamine for the treatment of this disease. The clinically relevant effects of ketamine administered in this way often long outlast the acute effects of this drug. However, intense (HFO-related) bursts of output from principal neurons have long been known to powerfully generate long-term synaptic plasticity [56]. Thus transient periods of HFO output from OB may induce long-lasting changes in functional connectivity of wider olfactory, cognitive and affective pathways in the brain.

## Electronic supplementary material

Supplementary 1. Power of HFO in the OB after saline injection

Supplementary 2. Time course of HFO before and after an anesthetic dose of ketamine

Supplementary 3. Power of HFO at baseline and post ketamine for 32 channel recordings

Supplementary Methods

APC
