# Supplementary figures and images for: The olfactory bulb is a source of high-frequency oscillations (130–180 Hz) associated with a subanesthetic dose of ketamine in rodents
Source: Neuropsychopharmacology. 2018 Aug 8;44(2):435–42. doi: 10.1038/s41386-018-0173-y (PMC6300534; doi:10.1038/s41386-018-0173-y)

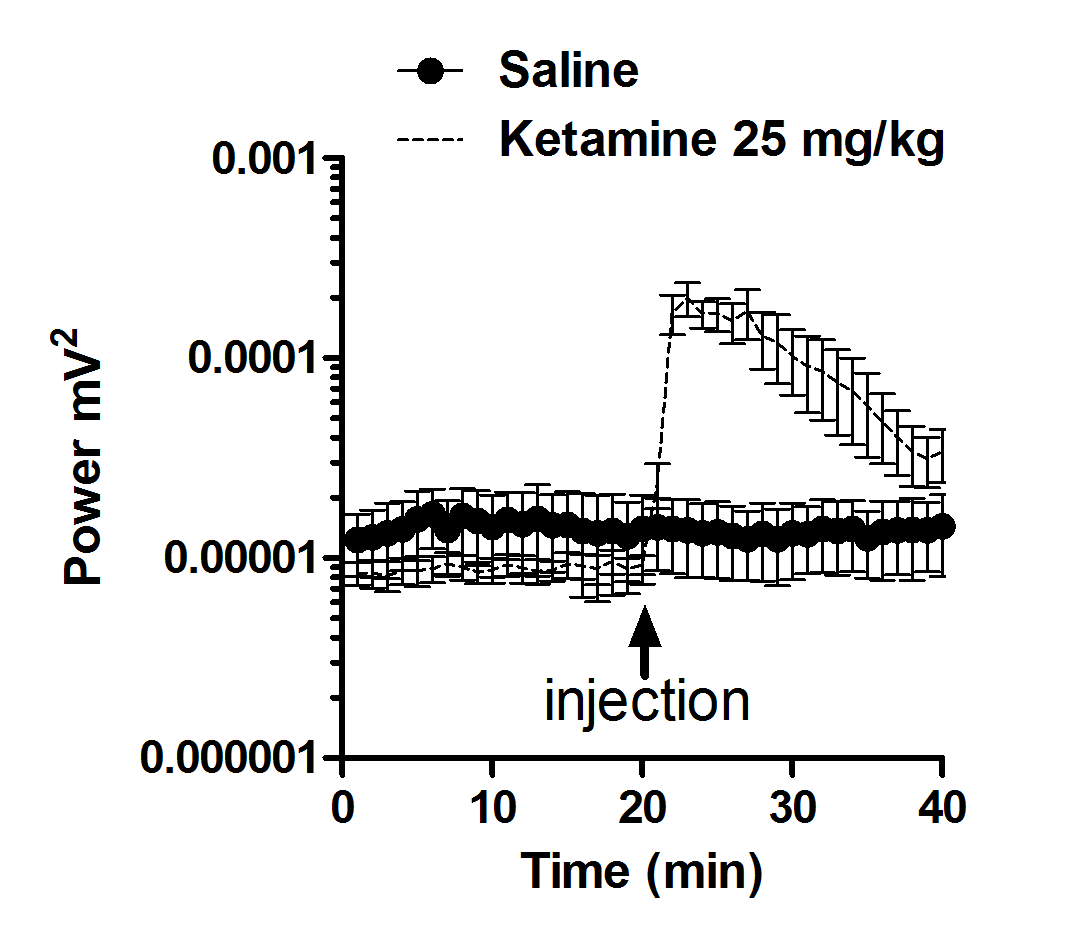

Supplement: Supplementary file 1 — Supplementary 1. Power of HFO in the OB after saline injection [file 41386_2018_173_MOESM1_ESM.tif]

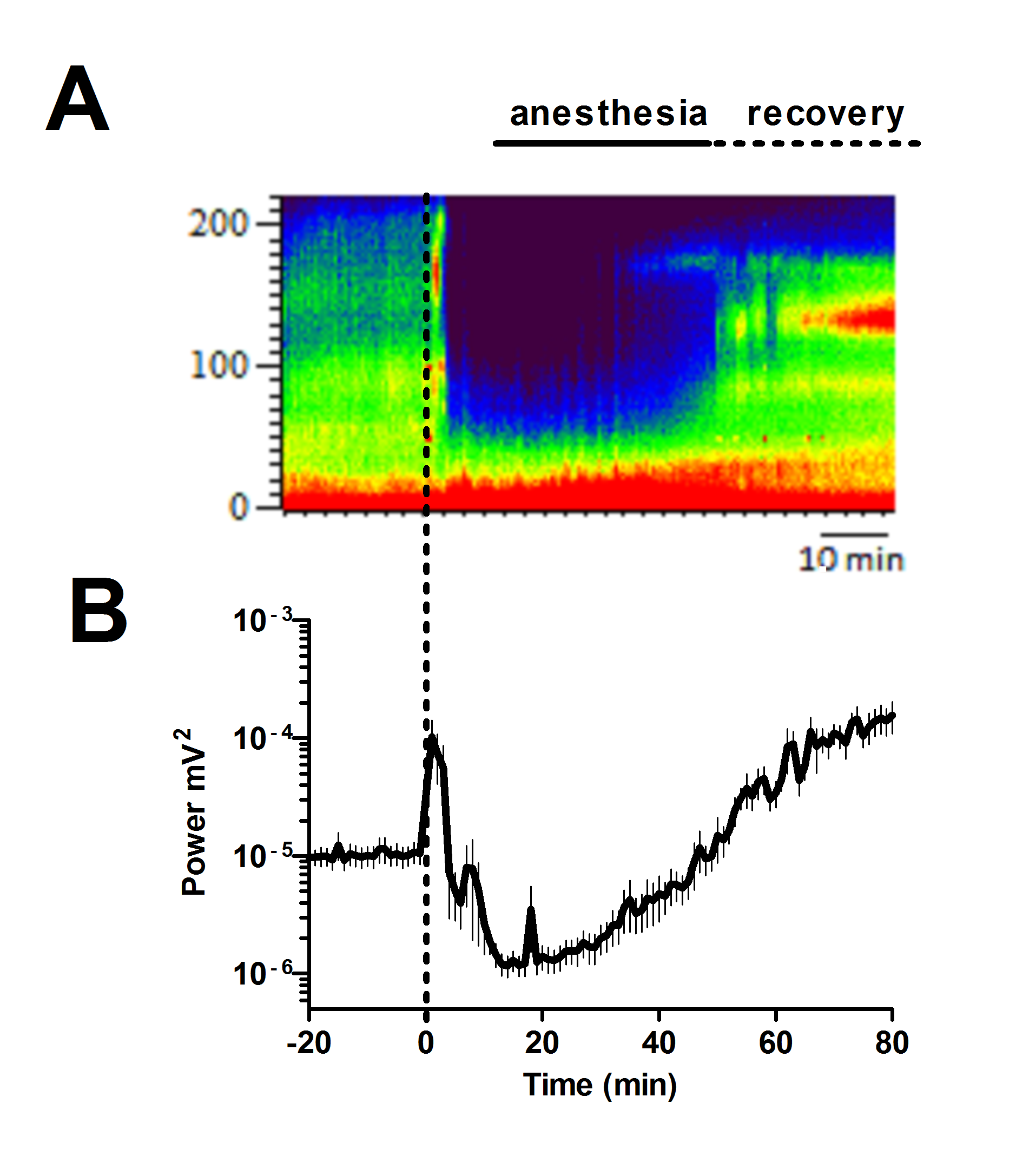

Supplement: Supplementary file 2 — Supplementary 2. Time course of HFO before and after an anesthetic dose of ketamine [file 41386_2018_173_MOESM2_ESM.tif]

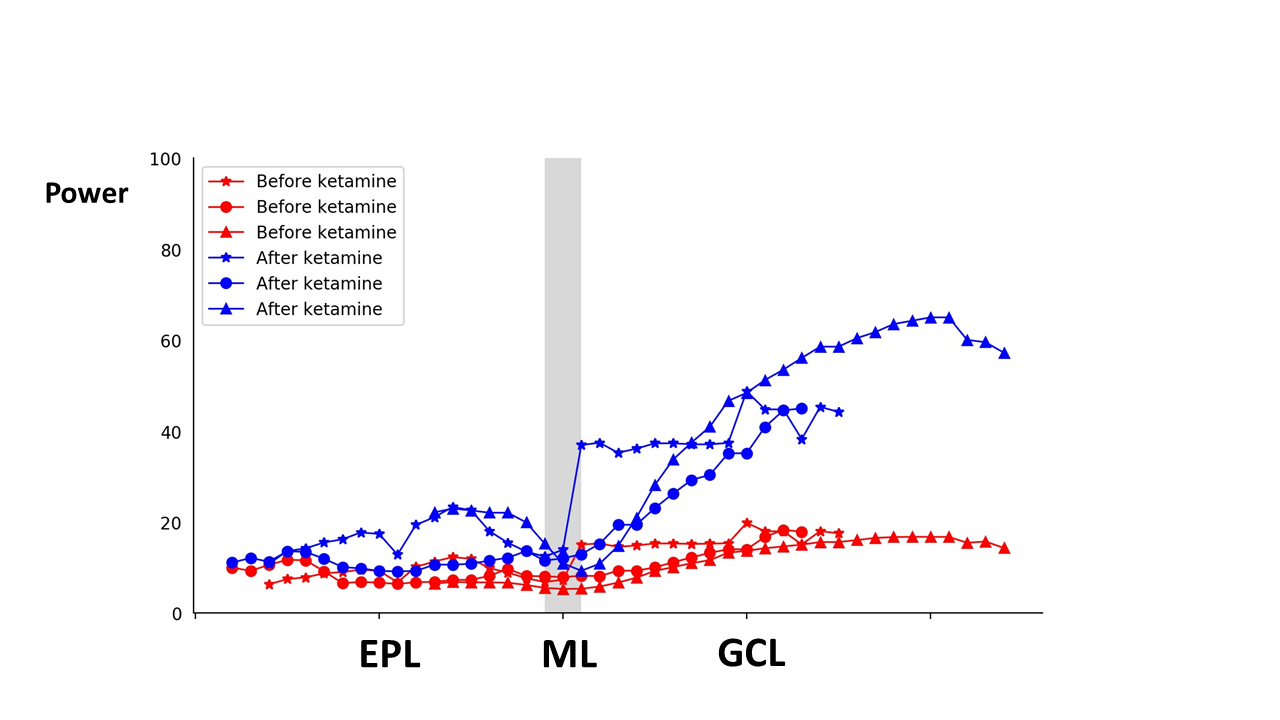

Supplement: Supplementary file 3 — Supplementary 3. Power of HFO at baseline and post ketamine for 32 channel recordings [file 41386_2018_173_MOESM3_ESM.tif]
