# Supplementary material for: The olfactory bulb is a source of high-frequency oscillations (130–180 Hz) associated with a subanesthetic dose of ketamine in rodents
Source: Neuropsychopharmacology. 2018 Aug 8;44(2):435–42. doi: 10.1038/s41386-018-0173-y (PMC6300534; doi:10.1038/s41386-018-0173-y)
Supplement: Supplementary file 4 — Supplementary Methods [file 41386_2018_173_MOESM4_ESM.docx]

**SUPPLEMENTARY MATERIALS AND METHODS**

**Surgery**

Experiments were performed on twenty male Wistar rats (250–350 g). Surgery was carried out under isoflurane anesthesia. In nine rats, electrode pairs made from twisted tungsten wire (125 mm, Science Products, Germany), insulated except at the tip, were implanted bilaterally in the olfactory bulb (AP=+7.5, ML=±0.5, DV=3-3.5 mm) and in the right nucleus accumbens subregion of the VS (AP=+1.6, ML=+0.8, DV=7 mm) according to co-ordinates of the stereotaxic atlas (Paxinos and Watson, 1986). In seven rats tungsten electrodes and 22 gauge stainless steel guides (Bilaney, Germany) were implanted bilaterally in the OB. Dummy internal cannulae were used to keep the guides patent. Tungsten electrodes were also implanted bilaterally in the VS. In four rats a silicon probe (Edge-10mm-20-177-H32_21mm, Neuronexus, USA), interelectrode distance 20 µm, was mounted on in-house manufactured microdrive 32 and implanted in the right OB. A screw posterior to the bregma was used as a reference/ground. A silver wire was used as ground/reference electrode connected to a screw posterior to the bregma. Five or six stainless steel anchoring screws were screwed into the skull and dental cement was applied to fix the headset in place. Rats were injected (i.p) with 10 mg/kg carprofen for 3 days after surgery. Rats were singly housed and kept on a 12 hour light-dark cycle. Food and water was provided *ad libitum.*

All necessary measures were taken to minimize pain or discomfort and the number of experimental animals used in this study. All experiments were conducted in accordance with the European community guidelines on the Care and Use of Laboratory Animals (86/609/EEC) and approved by a local ethics committee.

**Experimental procedure**

Experiments were performed from 9 AM and no later than 5 PM. One week after surgery rats were handled and habituated to the recording chamber (50x50x50 cm). Local field potentials (LFPs) were recorded through a JFET preamplifier, amplified x1000, filtered 0.01–1000 Hz and digitized at 1.6 kHz (AlphaLab SNR, AlphaOmega, Nazareth, Israel). For unit recording signals were filtered DC-11 kHz and sampled at 44 kHz. The signal was relayed through a commutator (Crist Instruments, USA) allowing the rat free movement inside the recording chamber.

Experiment 1: Baseline LFP recordings in the OB and VS were obtained 20 min before injection of ketamine or saline (n=9 rats). This experiment was carried out according to a Latin-square design, whereby each rat was ketamine 25 mg/kg (Sigma, UK). Rats were recorded for at least 45 min after injection. These rats were also administered with an anesthetic dose of ketamine 200 mg/kg (see experiment 3) and ketamine/xylazine as part of a separate study. The order of injections was pseudorandomised and at least 3 days separated injections.

Experiment 2: For infusion, stylets were removed and cannulae (28 gauge, Bilaney) that extended 2mm below the tip of the guide were inserted for 60s. This was followed by 60s infusion of muscimol (0.5 ug/0.5 ul) or saline (0.5 ul) and cannulae were left in place for a further 60s, the injection cannulea were removed and the stylets re-inserted to keep the guide patent. Immediately after infusion rats were reconnected and 25 mg/kg ketamine injected i.p. Experiments were conducted in a Latin square design, whereby one side received infusion of muscimol and the opposite side saline. Infusions were separated by at least 3 days.

Experiment 3: During the recovery phase of ketamine anesthesia 200 mg/kg (but not during deep anesthesia) HFO is visible in the LFP spectra of OB and VS recordings. Rats are manageable during the initial recovery stage. LFP’s were recorded in the OB and NAc. One naris was occluded by using a cotton bud to press a soft rubber base against the nostril. Naris blockade lasted for approximately 100 seconds. This procedure was also repeated for the naris on the opposite side. Rats for this study were also used in Experiment 1. There rats were also injected with saline and ketamine/xylazine as part of a separate study.

Experiment 4: 32 channel silicon probes (A1x32-Edge-10mm-20-177-H32_21mm) were gradually advanced over several recording days until they reached a depth of around 3-4 mm. Recordings were made before and after injection of ketamine 25 mg/kg at different depths with 2-4 days separating each recording session.

Histology: At the end of the study skulls were post fixed in 4% paraformaldehyde for 3-4 days. The brains were then dissected and fixed for a further 3 days in 4% paraformaldehyde followed by 10% and 40% sucrose solution. The brains were sectioned (40 microns) using a cryostat and mounted on gelatin coated microscope slides. The location of the electrode tips was determined on Cresyl violet or Hoechst stained sections.

**Data Analysis**

Data were saved and analysed offline. Digitized LFP signals were visually inspected and data segments with artefacts were removed manually and discarded for further analysis. These were rare occurrences such as obvious movement artefacts which resulted in large deflections in the signal.

FFT analysis: Mean power spectra of the LFP were carried out on successive 60-s data blocks using a fast Fourier transform and the dominant power of HFO (130-180 Hz) calculated. FFT’s were calculated using an in-built script in Spike 2 (CED, United Kingdom). Coherence between the raw LFP recorded in that OB and VS was calculated for a 60 sec window shortly after injection of ketamine and compared to 60 sec at the end of baseline. Analysis was carried out using a script available from the CED website http:// [www.ced.co.uk/upu.shtml](http://www.ced.co.uk/upu.shtml). Waveform correlation between the 130-180 Hz band pass filtered signals in the OB and VS were carried out for 60 sec shortly after ketamine.

Granger Causality: Granger 'causality' was computed over a series of orders (1 - 10) for frequencies ranging from 1 - 350 Hz. The LFPs were split into 500 ms sections overlapped by 10 ms to create trials over which the results were averaged, which was then repeated for each 'N'. This was performed using a 50 ms window with one_bi_ga.m from the BSMART toolbox (SHIS UT-Houston, Houston, TX 77030, USA) in MATLAB.

Current source density analysis: We estimated current source density (CSD) in one spatial dimension as a function of time. The plot in Figure 4C shows sinks (blue) and sources (red) of the band-pass (140-200 Hz, HFO) filtered olfactory bulb activity. The problem of estimating current source is non-trival issue as there are many sources and sinks distribution that fits to recorded potential (Potworowski et. al. 2012). For our analysis we used kernel (kCSD) inverse method to calculate CSD. To estimate normalization parameter and base function radius we applied L-curve method. Mean CSD’s were triggered on the trough of HFO events in the most dorsal electrode contact, since the amplitude of HFO tended to be larger at more dorsal contacts.

Phase analysis: Phase differences were calculated for the 130-180 Hz filtered signal with respect to the most dorsal contact (HFO has large amplitude). For relative phase analysis we performed fast fourier transform (FFT) for 25 ms time window of the 140-200 Hz filtered signal starting from the trough of the HFO in the most dorsal contact. To estimate phase of the oscillations we picked imaginary part of the FFT for ~180 Hz.

Multi-unit activity: Raw LFP’s were digitally filtered using a >500 Hz high-pass filter. The continuous electrophysiological recordings were divided into ~200 sweeps (depending on animal) of 50 ms time duration. We choose parts of the signal with high amplitude High Frequency Oscillations (HFO) triggering from trough and taking +- 25 ms of the waveform. For HFO trough detection signal was filtered 140 – 200 Hz using bandpass Butterworth third order filter. Raster plot of the multi-unit activity used threshold of 1.5 and 3 standard deviation estimated from 500 Hz high-pass Butterworth third order filtered signal. To reduce noise artefacts we took first principal component for the recordings from the mitral and EPL layers.
