# Supplementary material for: The olfactory bulb is a source of high-frequency oscillations (130–180 Hz) associated with a subanesthetic dose of ketamine in rodents
Source: Neuropsychopharmacology. 2018 Aug 8;44(2):435–42. doi: 10.1038/s41386-018-0173-y (PMC6300534; doi:10.1038/s41386-018-0173-y)
Supplement: Supplementary file 5 — APC [file 41386_2018_173_MOESM5_ESM.docx]

Supplementary 1. Power of HFO in the OB after saline injection. Mean power of HFO before and

after injection of saline (n=6 rats). For comparison the dotted line shows the increase in HFO power

induced by injection of ketamine.

Supplementary 2. Time course of HFO before and after an anesthetic dose of ketamine. HFO power

is attenuated during 200 mg/kg ketamine anesthesia, but increases during the recovery stage. Naris

experiments used in Figure 3 were carried out during the initial part of the recovery phase.

Supplementary 3. Power of HFO at baseline and post ketamine for 32 channel recordings. Power of

HFO across all contacts (32 channel linear array) at before and after injection of 25 mg/kg ketamine.
